# No Association between Vitamin D and Weight Gain: A Prospective, Population-Based Study

**DOI:** 10.3390/nu14153185

**Published:** 2022-08-03

**Authors:** Pollyanna Patriota, Serge Rezzi, Idris Guessous, Pedro Marques-Vidal

**Affiliations:** 1 Swiss Nutrition and Health Foundation, 1066 Epalinges, Switzerland; pollypatriota2@gmail.com (P.P.); serge.rezzi@nutritionhealthfoundation.ch (S.R.); 2 Division of Primary Care Medicine, Department of Primary Care Medicine, Geneva University Hospitals, 1205 Geneva, Switzerland; idris.guessous@hcuge.ch; 3 Department of Medicine, Internal Medicine, Lausanne University Hospital and University of Lausanne, 46 rue du Bugnon, 1011 Lausanne, Switzerland

**Keywords:** vitamin D, epidemiology, weight gain

## Abstract

Background: The association between vitamin D and weight gain remains controversial due to important limitations in the studies. We investigated the relationship between vitamin D levels and 5 and 10 years of weight and waist circumference change in a population-based prospective cohort study. Methods: Prospective study including participants aged between 35 and 75 years living in the city of Lausanne, Switzerland. Weight and waist change at 5- and 10-year follow-up were assessed according to baseline vitamin D status (normal, insufficiency and deficiency). Results: A total of 3638 participants (47.9 % women, mean age 51.6 ± 10.4 years) were included for the 5-year follow-up. No association was found between vitamin D categories and weight change, multivariate-adjusted average ± standard error: 1.6 ± 0.3, 1.5 ± 0.2 and 1.2 ± 0.1 kg for normal, insufficiency and deficiency, respectively, *p* = 0.159. For waist change, the corresponding values were 3.3 ± 0.4, 3.3 ± 0.2 and 3.4 ± 0.2 cm, *p* = 0.792. For the 10-year follow-up, data from 2999 participants (45.8% women, mean age 50.8 ± 10.3 years) were used. No association was found for weight 2.3 ± 0.4, 2.3 ± 0.2 and 2.0 ± 0.2 kg, *p* = 0.588, or for waist 3.7 ± 0.4, 3.6 ± 0.3 and 4.2 ± 0.2 cm for normal, insufficiency and deficiency, respectively, *p* = 0.259. Conclusion: No association between vitamin D status and weight or waist gain at 5- and 10-year follow-up was found.

## 1. Introduction

25-hydroxyvitamin D (25(OH)D) (from now on indicated as “vitamin D”) levels are inversely associated with adiposity markers [1,2,3,4]. Although a causal relationship between these two outcomes has not yet been fully elucidated [5,6], a possible mechanism is a decreased bioavailability of vitamin D in individuals with obesity [7,8]. The inverse association between obesity and vitamin D levels could also be explained by lifestyle differences between normal weight and individuals with obesity [5,6], suggesting that vitamin D can be a biomarker of a healthy lifestyle and not an independent risk factor for obesity [9]. 

The associations between vitamin D levels and weight change have been less well studied. A study found that higher serum vitamin D levels were associated with a reduced risk of weight gain in normal weight adults [10]. Another study reported that subjects who gained ≥5% weight had lower vitamin D levels than subjects who lost >5% weight [11]. Vogt et al. found that vitamin D levels were not associated with overall weight change or body fat loss, and that higher vitamin D levels were associated with a lower likelihood of gaining body fat in women but not in men [12]. Overall, the association between vitamin D and weight gain remains controversial due to important limitations in the studies.

In this study, we aimed to assess the associations between vitamin D levels and changes in weight, body mass index (BMI) and waist circumference, at 5- and 10-year follow-up in a general adult population.

## 2. Participants and Methods

### 2.1. Study Design

The CoLaus (Cohorte Lausannoise) study is a population-based prospective study assessing the clinical, biological, and genetic determinants of cardiovascular disease aged 35 to 75 years at baseline, living in the city of Lausanne, Switzerland [13]. In each survey, participants answered questionnaires, underwent a clinical examination and blood samples were drawn for analyses. Recruitment began in June 2003 and ended in May 2006 and included 6733 participants. The first follow-up was performed between April 2009 and September 2012 (N = 5064 participants) and the second follow-up between May 2014 and April 2017 (N = 4881). Median follow-up time was 5.4 (average 5.6, range 4.5–8.8) years for the first follow-up and 10.7 (average 10.9, range 8.8–13.6) years for the second follow-up.

### 2.2. Anthropometry

Within each survey, anthropometric measurements were conducted using a standard methodology. Body weight and height were measured with participants barefoot and in light indoor clothes. Body weight was measured in kilograms to the nearest 100 g using a Seca^®^ scale (Hamburg, Germany). Height was measured to the nearest 5 mm using a Seca^®^ (Hamburg, Germany) height gauge. Weight change was defined as the difference between the last and the first visit and three metrics were used: (1) as a continuous variable; (2) categorized based on % weight change into losers (loss >5% of initial weight), gainers (gain >5% of initial weight) and maintainers (other) [14,15], and (3) categorized based on absolute values into losers (loss >5 kg), gainers (gain >5 kg) and maintainers (other) [16].

Waist circumference (WC) was measured mid-way between the lowest rib and the iliac crest, waist change was defined as the difference between the last and the first visit, and three metrics were used: (1) as a continuous variable; (2) categorized based on absolute values into losers (loss >5 cm), gainers (gain >5 cm) and maintainers (other).

Body mass index (BMI) was computed and the difference between the last and the first visit was used as a continuous variable.

### 2.3. Vitamin D Levels

Blood samples for vitamin D measurement were collected when the participant attended the baseline (2003–2006) visit. Vitamin D was assessed through an ultra-HPLC tandem-MS system. The calibrators, 3Plus1 Multilevel Serum Calibrator Set 25-OH-Vitamin D3/D2 (ChromoSystems), were standardized against the National Institute of Standards and Technology 972 reference material. The interday CV% was 4.6% at 40 nmol/L [17]. Vitamin D levels were further categorized as normal (≥75 nmol/L or 30 ng/mL), insufficiency (50 to 74 nmol/L or 20 to 29 ng/mL) and deficiency (<50 nmol/L or 20 ng/mL) [18]. Hypovitaminosis D was defined for vitamin D levels < 75 nmol/L or 30 ng/mL, encompassing insufficiency plus deficiency [19].

### 2.4. Other Covariates

Educational level was categorized into university, high school, apprenticeship, and mandatory. Nationality was categorized into being born in Switzerland or not. Smoking status was self-reported and categorized as never, former, and current. Physical activity was considered if the participant reported performing at least twice a week a minimum of 20 min of leisure-time physical activity. No information regarding dietary intake or sun exposure was collected.

### 2.5. Exclusion Criteria

Participants were excluded if they (1) lacked vitamin D data; (2) had no anthropometric measurements; (3) missed any covariate (education, smoking, BMI, or physical activity); (4) reported being on a slimming diet; (5) had no follow-up; and (6) reported taking any supplement susceptible of containing vitamin D.

### 2.6. Statistical Analysis

Statistical analyses were performed separately for each follow-up period using Stata version 16.1 for Windows (Stata Corp, College Station, TX, USA). Descriptive results were expressed as number of participants (percentage) for categorical variables and as average ± standard deviation or median [interquartile range] for continuous variables.

Bivariate comparison of weight and waist change between vitamin D categories was performed using ANOVA and the results were expressed as average ± standard deviation. Multivariate analysis of weight and waist change between vitamin D categories was performed overall and by sex using ANOVA adjusted for age (continuous), nationality (Swiss, other), month (January to December), smoking categories (never, former, current) and physical activity (yes, no). For women, a further adjustment on menopausal status (yes, no) was performed. For the overall analysis, adjustment on sex was also performed. Results were expressed as adjusted average ± standard error. For both bivariate and multivariate analyses, a test for linear trend of the effect of vitamin D categories (normal, insufficiency, deficiency) was applied.

The associations between changes in anthropometric markers and vitamin D levels (as a continuous variable) were assessed using Spearman nonparametric correlation and multivariate linear regression, adjusting for the same variables as above. The results of the multivariate analysis were expressed as beta coefficients.

For all analyses, statistical significance was considered for a two-sided test with *p* < 0.05.

### 2.7. Ethical Statement

The institutional Ethics Committee of the University of Lausanne, which afterwards became the Ethics Commission of Canton Vaud (www.cer-vd.ch), approved the baseline CoLaus study (reference 16/03, decisions of 13 January and 10 February 2003). The approval was renewed for the first (reference 33/09, decision of 23 February 2009) and second (reference 26/14, decision of 11 March 2014) follow-ups. The approval for the entire CoLaus|PsyCoLaus study was confirmed in 2021 (reference PB_2018-00038, 239/09, decision of 21 June 2021). The full decisions of the CER-VD can be obtained from the authors upon request. The study was performed in agreement with the Helsinki declaration and its former amendments, and in accordance with the applicable Swiss legislation. All participants gave their signed informed consent before entering the study.

## 3. Results

### 3.1. Participants Selection and Characteristics

**Figure 1:**
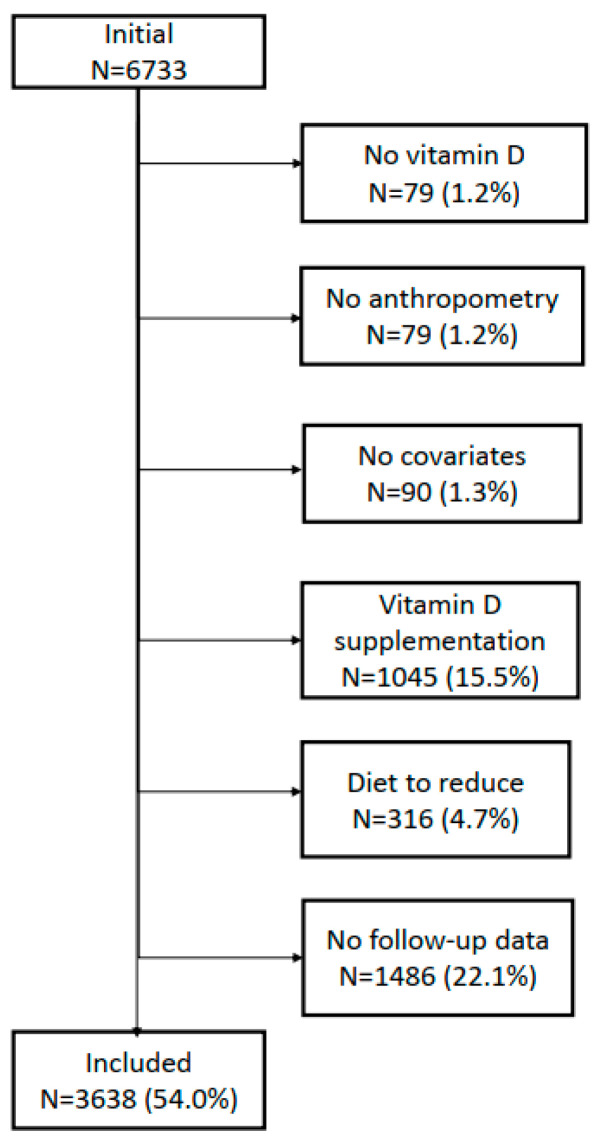
Selection of participants for the 5-year follow-up, CoLaus|PsyCoLaus study, Lausanne, Switzerland.

**Figure 2:**
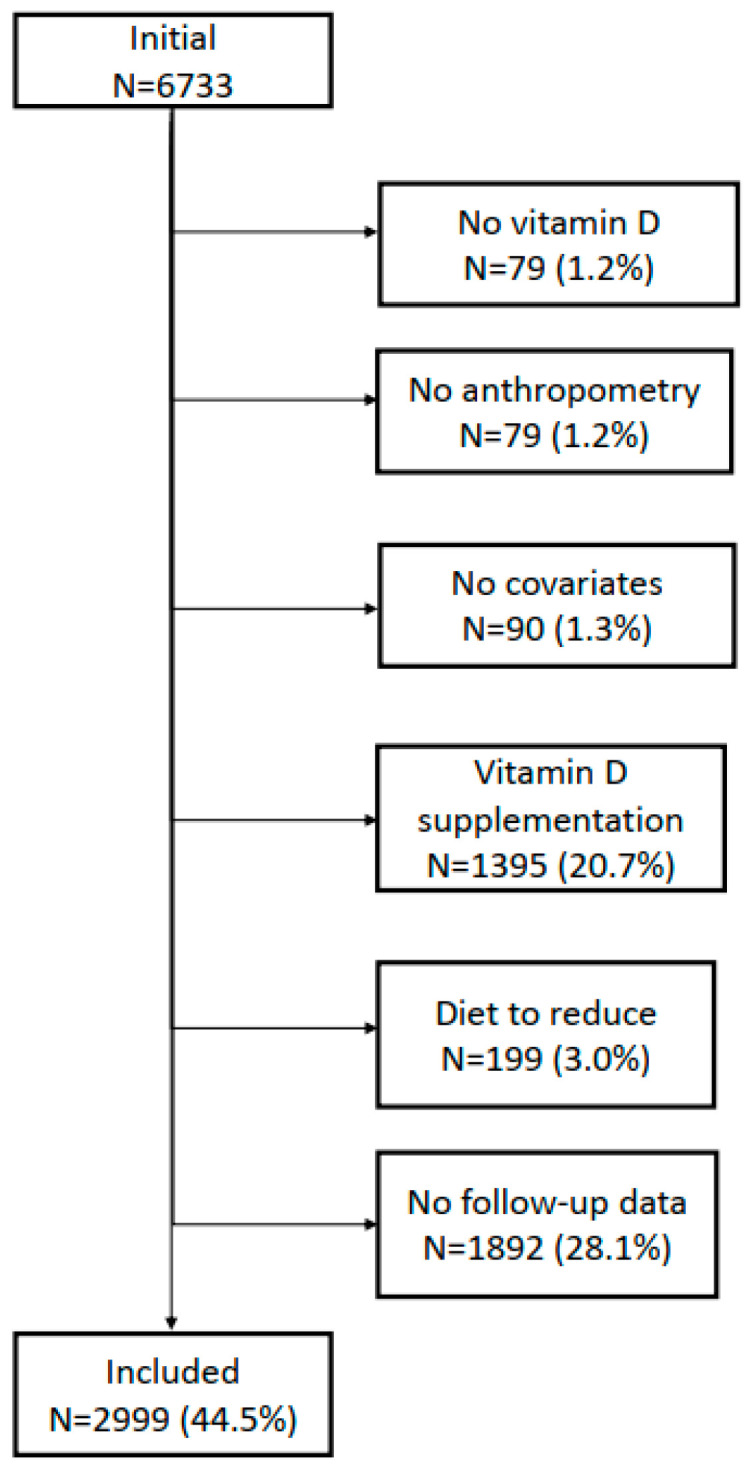
Selection of participants for the 10-year follow-up, CoLaus|PsyCoLaus study, Lausanne, Switzerland.

The selection procedure of the participants for the 5- and 10-year follow-ups is indicated in Figure 1 and Figure 2, respectively. For the 5-year follow-up, 54% of the initial 6733 participants were included, while for the 10-year follow-up this percentage was 44.5%.

The characteristics between excluded and included participants of 5- and 10-year follow-ups are shown in the Supplementary Table S1. Excluded participants were older, more frequently women, of lower education, less frequently Swiss-born, and physically active.

### 3.2. Associations between Vitamin D Levels and Weight Changes at 5 and 10-Year Follow-Up

The prevalence of normal, insufficient and deficient vitamin D levels at baseline was 11%, 30% and 59%, respectively.

The baseline characteristics of the participants who completed the 5-year follow-up according to sex are indicated in Supplementary Table S2. Women were less educated, were more frequently non-smokers, with normal weight or underweight than men. Women presented more frequently with abdominal obesity, while no difference was found for vitamin D status. The 5-year changes in weight, BMI and waist according to vitamin D categories are indicated in Table 1, overall and by sex. No differences between vitamin D categories were found regarding weight, BMI or waist changes in bivariate or multivariate analyses.

The baseline characteristics of the participants who completed the 10-year follow-up according to sex are indicated in Supplementary Table S2. Women were less educated, were more frequently non-smokers, with normal weight or underweight than men. Women presented more frequently with abdominal obesity, while no differences were found for vitamin D status. The 10-year changes in weight, BMI and waist according to vitamin D categories are indicated in Table 2, overall and by sex. No differences between vitamin D categories regarding were found regarding weight, BMI or waist changes in bivariate or multivariate analyses.

The results of the associations between vitamin D levels as a continuous variable and 5- and 10-year changes in anthropometric markers are indicated in Table 3. No consistent significant association was found.

## 4. Discussion

In this large prospective study, no association was found between vitamin D levels and weight, BMI or waist change. 

### 4.1. Prevalence of Vitamin D Deficiency and Insufficiency

The prevalence of normal, insufficient and deficient vitamin D levels at baseline was 11%, 30% and 59%, respectively, leading to an 89% prevalence of hypovitaminosis D. Those values are comparable to a French study [20], where 80.3% of participants aged between 18 and 89 years presented with hypovitaminosis D, and higher than a Swiss study [21], where the prevalence of hypovitaminosis was 74.7%. A review focusing on South European countries [22] reported a prevalence of hypovitaminosis D ranging between 18% and 75%. A study focusing mostly on North European countries reported a prevalence of vitamin D deficiency ranging between 46% and 56% [23]. Overall, our results are slightly higher compared with other studies conducted in apparently healthy populations.

### 4.2. Associations between Vitamin D Levels and Weight, Body Mass Index or Waist Changes

In this study, after adjustment for potential confounders, no association between vitamin D status and weight, BMI or waist gain was found, neither at 5-year nor at 10-year follow-up. Similarly, no significant associations were found for all anthropometric markers when vitamin D levels were used as a continuous variable. There are conflicting data regarding the association between vitamin D levels and weight or waist gain. Our results are in agreement with a study conducted in Southern Germany, where no association was found between vitamin D levels and overall weight or body fat changes [12]. Similarly, a study including 10,898 participants from three different cohorts found no association between vitamin D and changes in measures of adiposity [24]. Conversely, an inverse association between vitamin D levels and weight or waist gain was reported among Finnish men (but no Finnish women) [9] and in a Norwegian study [10]. Similarly, a study conducted in Spain found that vitamin D deficiency was significantly associated with an increased risk of developing obesity in the next 4 years [25]. Finally, a study conducted among American women found no association between vitamin D levels and weight change overall, but reported a significant interaction between vitamin D levels and weight change category: in women who gained weight, those with low vitamin D levels gained more weight than those with higher vitamin D levels [26]. Overall, our results add further strength to the supporters of the hypothesis that vitamin D does not influence weight or waist gain.

### 4.3. Strengths and Limitations

This study was conducted in a representative sample of the Lausanne population, the sample size was larger than several other studies on the same topic [9,10,25], and a long follow-up was available.

Some limitations should also be acknowledged. Firstly, the sample was mainly composed of Caucasian participants living in a single Swiss city. Hence, our findings might not be generalized to other settings or ethnicities. Secondly, we could not adjust for other potential confounders such as diet and sun exposure, although we did adjust for seasonality. Still, it should be noted that the other studies adjusted neither for diet (besides alcohol consumption), nor for sunshine [9,10,12,24,25,26], but most (except [12] and [26]) adjusted for season. Moreover, we did not take into account the fact that some participants might have started vitamin D containing supplements during the follow-up [27], or might have omitted them at baseline. Still, and as reported in the previous study, the percentage of participants starting supplements was small (11.7%) and mirrored by a similar percentage of participants discontinuing them (12%). Hence, the effect of starting vitamin supplements during the follow-up on weight or waist gain is likely modest. Finally, a single vitamin D measurement was performed, and it might not fully reflect the vitamin D status of an individual; still, most studies used a single measurement [10,12,28] and performing several measurements would have been impractical and very expensive in an epidemiological setting.

## 5. Conclusions

We found no association between vitamin D status and weight, BMI or waist gain at 5- and 10-year follow-up in an urban Swiss population.

## Figures and Tables

**Table 1:** Bivariate and multivariate comparisons of 5-year changes in weight and waist according to baseline vitamin D categories, overall and by sex, CoLaus|PsyCoLaus study, Lausanne.

	Overall			Women			Men		
	N	Bivariate	Multivariate	N	Bivariate	Multivariate	N	Bivariate	Multivariate
Weight (kg)									
Vitamin D categories									
Normal	396	1.5 ± 4.4	1.6 ± 0.3	179	1.6 ± 3.6	1.7 ± 0.4	217	1.5 ± 4.9	1.5 ± 0.3
Insufficiency	1106	1.5 ± 4.4	1.5 ± 0.2	541	1.6 ± 4.4	1.6 ± 0.2	565	1.4 ± 4.3	1.3 ± 0.2
Deficiency	2136	1.2 ± 5.3	1.2 ± 0.1	1004	1.2 ± 5.6	1.2 ± 0.2	1132	1.2 ± 4.9	1.3 ± 0.1
*p*-value for trend		0.224	0.159		0.300	0.254		0.499	0.586
Weight (% initial)									
Vitamin D categories									
Normal	396	2.3 ± 5.8	2.5 ± 0.3	179	2.6 ± 5.9	2.7 ± 0.6	217	2.1 ± 5.8	2.1 ± 0.4
Insufficiency	1106	2.2 ± 6.1	2.1 ± 0.2	541	2.6 ± 6.7	2.6 ± 0.3	565	1.8 ± 5.4	1.6 ± 0.2
Deficiency	2136	1.8 ± 6.9	1.8 ± 0.1	1004	2.0 ± 7.8	1.9 ± 0.2	1132	1.6 ± 5.9	1.7 ± 0.2
*p*-value for trend		0.124	0.062		0.264	0.208		0.272	0.352
Waist (cm)									
Vitamin D categories									
Normal	396	3.1 ± 6.0	3.3 ± 0.4	179	4.1 ± 6.2	4.2 ± 0.6	217	2.3 ± 5.7	2.2 ± 0.4
Insufficiency	1106	3.3 ± 6.4	3.3 ± 0.2	541	4.5 ± 7.0	4.6 ± 0.3	565	2.1 ± 5.4	2.0 ± 0.3
Deficiency	2136	3.4 ± 7.2	3.4 ± 0.2	1004	5.0 ± 8.1	5.0 ± 0.2	1132	1.9 ± 6.0	2.0 ± 0.2
*p*-value for trend		0.483	0.792		0.117	0.234		0.319	0.603
BMI (kg/m^2^)									
Vitamin D categories									
Normal	396	0.7 ± 1.5	0.7 ± 0.1	179	0.7 ± 1.4	0.8 ± 0.2	217	0.6 ± 1.6	0.6 ± 0.1
Insufficiency	1106	0.6 ± 1.5	0.6 ± 0.1	541	0.7 ± 1.7	0.7 ± 0.1	565	0.5 ± 1.4	0.5 ± 0.1
Deficiency	2136	0.5 ± 1.9	0.5 ± 0.1	1004	0.6 ± 2.1	0.6 ± 0.1	1132	0.5 ± 1.6	0.5 ± 0.1
*p*-value for trend		0.122	0.111		0.243	0.240		0.295	0.450

Results are expressed as mean standard ± deviation for bivariate analyses or as adjusted mean ± standard error for multivariate analyses. Statistical analysis using ANOVA. Multivariate analysis adjusting for age (continuous), nationality (Swiss, other), month (January to December), smoking categories (never, former, current) and physical activity (yes, no). For women, adjustment on menopause (yes, no) was also performed. For the overall analysis, adjustment on sex (men, women) was also performed.

**Table 2:** Bivariate and multivariate comparisons of 10-year changes in weight and waist according to baseline vitamin D categories, overall and by sex, CoLaus|PsyCoLaus study, Lausanne.

	Overall			Women			Men		
	N	Bivariate	Multivariate	N	Bivariate	Multivariate	N	Bivariate	Multivariate
Weight (kg)									
Vitamin D categories									
Normal	349	1.9 ± 5.4	2.3 ± 0.4	153	2.1 ± 4.9	2.1 ± 0.5	196	1.7 ± 5.7	2.3 ± 0.5
Insufficiency	919	2.3 ± 6.0	2.3 ± 0.2	437	2.6 ± 5.8	2.7 ± 0.3	482	2.0 ± 6.2	1.9 ± 0.3
Deficiency	1731	2.1 ± 6.9	2.0 ± 0.2	783	2.1 ± 7.4	2.1 ± 0.2	948	2.1 ± 6.5	2.1 ± 0.2
*p*-value for trend		0.631	0.588		0.956	0.940		0.478	0.719
Weight (% initial)									
Vitamin D categories									
Normal	349	2.9 ± 7.4	3.4 ± 0.5	153	3.4 ± 7.7	3.5 ± 0.8	196	2.4 ± 7.1	3.1 ± 0.6
Insufficiency	919	3.3 ± 8.2	3.2 ± 0.3	437	4.1 ± 8.8	4.1 ± 0.4	482	2.6 ± 7.6	2.4 ± 0.3
Deficiency	1731	3.0 ± 8.9	2.9 ± 0.2	783	3.3 ± 10.0	3.3 ± 0.3	948	2.7 ± 7.8	2.7 ± 0.3
*p*-value for trend		0.810	0.375		0.895	0.812		0.635	0.594
Waist (cm)									
Vitamin D categories									
Normal	349	3.7 ± 6.5	3.7 ± 0.4	153	3.8 ± 7.1	3.4 ± 0.7	196	3.7 ± 6.0	3.7 ± 0.5
Insufficiency	919	3.8 ± 7.1	3.6 ± 0.3	437	4.5 ± 7.4	4.3 ± 0.4	482	3.1 ± 6.7	3.0 ± 0.3
Deficiency	1731	4.1 ± 7.8	4.2 ± 0.2	783	4.7 ± 8.8	4.9 ± 0.3	948	3.6 ± 6.8	3.7 ± 0.2
*p*-value for trend		0.393	0.259		0.208	0.054		0.885	0.954
BMI (kg/m^2^)									
Vitamin D categories									
Normal	349	1.0 ± 1.9	1.1 ± 0.1	153	1.1 ± 1.9	1.1 ± 0.2	196	0.8 ± 1.9	1.0 ± 0.2
Insufficiency	919	1.1 ± 2.1	1.1 ± 0.1	437	1.3 ± 2.2	1.3 ± 0.1	482	0.9 ± 1.9	0.8 ± 0.1
Deficiency	1731	1.0 ± 2.4	1.0 ± 0.1	783	1.1 ± 2.8	1.1 ± 0.1	948	0.9 ± 2.1	0.9 ± 0.1
*p*-value for trend		0.712	0.657		0.895	0.914		0.706	0.673

BMI, body mass index. Results are expressed as mean standard ± deviation for bivariate analyses or as adjusted mean ± standard error for multivariate analyses. Statistical analysis using ANOVA. Multivariate analysis adjusting for age (continuous), nationality (Swiss, other), month (January to December), smoking categories (never, former, current) and physical activity (yes, no). For women, adjustment on menopause (yes, no) was also performed. For the overall analysis, adjustment on sex (men, women) was also performed.

**Table 3:** Bivariate and multivariate associations between 5- and 10-year changes in weight, waist and body mass index according to baseline vitamin D levels, overall and by sex, CoLaus|PsyCoLaus study, Lausanne.

	5-Year Changes					10-Year Changes				
	N	Bivariate	*p*-Value	Multivariate	*p*-Value	N	Bivariate	*p*-Value	Multivariate	*p*-Value
Weight (kg)										
Overall	3638	0.016	0.335	0.021	0.441	2999	−0.019	0.292	−0.025	0.394
Women	1724	0.015	0.529	0.020	0.473	1373	−0.023	0.387	−0.027	0.360
Men	1914	0.017	0.468	0.005	0.851	1626	−0.018	0.481	−0.006	0.842
Weight (% initial)										
Overall	3638	0.035	0.037	0.048	0.079	2999	0.002	0.904	0.024	0.412
Women	1724	0.039	0.102	0.047	0.087	1373	0.004	0.898	0.023	0.443
Men	1914	0.030	0.193	0.026	0.359	1626	−0.001	0.973	−0.005	0.874
Waist (cm)										
Overall	3633	−0.010	0.545	−0.012	0.658	2991	−0.023	0.222	−0.065	0.034
Women	1724	−0.039	0.104	−0.014	0.625	1371	−0.046	0.091	−0.065	0.034
Men	1909	0.017	0.455	0.006	0.827	1620	−0.005	0.853	−0.015	0.619
BMI (kg/m^2^)										
Overall	3637	0.035	0.035	0.024	0.394	2994	−0.022	0.232	−0.014	0.644
Women	1723	0.024	0.315	0.023	0.413	1371	−0.031	0.251	−0.015	0.599
Men	1914	0.044	0.056	0.019	0.490	1623	−0.017	0.488	−0.031	0.302

BMI, body mass index. Results are expressed as Spearman correlation coefficient for bivariate analyses or as adjusted beta slope for multivariate analyses. Multivariate analysis adjusting for age (continuous), nationality (Swiss, other), month (January to December), smoking categories (never, former, current) and physical activity (yes, no). For women, adjustment on menopause (yes, no) was also performed. For the overall analysis, adjustment on sex (men, women) was also performed.

## Data Availability

The CoLaus|PsyCoLaus cohort data used in this study cannot be fully shared as they contain potentially sensitive patient information. As discussed with the competent authority, the Research Ethic Committee of the Canton of Vaud, transferring or directly sharing t data would be a violation of the Swiss legislation aiming to protect the personal rights of participants. Non-identifiable, individual-level data are available for interested researchers, who meet the criteria for access to confidential data sharing, from the CoLaus Datacenter (CHUV, Lausanne, Switzerland). Instructions for gaining access to the CoLaus data used in this study are available at https://www.colaus-psycolaus.ch/professionals/how-to-collaborate/ (accessed on 28 July 2022).

## Supplementary Materials

The following supporting information can be downloaded at: https://www.mdpi.com/article/10.3390/nu14153185/s1, Table S1: comparison of the characteristics at baseline (2003–2006) between excluded and included participants for each follow-up, CoLaus|PsyCoLaus study, Lausanne; Table S2: comparison of the characteristics at baseline (2003-2006) of participants who completed the first and the second follow-ups, according to gender, CoLaus|PsyCoLaus study, Lausanne.
