# Supplementary material for: No Association between Vitamin D and Weight Gain: A Prospective, Population-Based Study
Source: Nutrients. 2022 Aug 3;14(15):3185. doi: 10.3390/nu14153185 (PMC9370694; doi:10.3390/nu14153185)
Supplement: Supplementary file 1 [file nutrients-14-03185-s001.zip › nutrients-1823907-supplementary.pdf]

Supplementary information

Supplementary table S1: comparison of the characteristics at baseline (2003-2006) between excluded and included participants for each follow-up, CoLaus|PsyCoLaus study, Lausanne.

	**5-year**			**10-year**		
	**Included**	**Excluded**	**P-value**	**Included**	**Excluded**	**P-value**
Sample size	**3638**	**3095**		**2999**	**3720**	
Age (years)	51.6 ± 10.4	53.8 ± 11.0	<0.001	50.8 ± 10.3	54.1 ± 10.8	<0.001
Women (%)	1724 (47.4)	1820 (58.8)	<0.001	1373 (45.8)	2171 (58.1)	<0.001
Born in Switzerland (%)	2290 (63.0)	1741 (56.3)	<0.001	1880 (62.7)	2151 (57.6)	<0.001
Education (%)			<0.001			<0.001
University	777 (21.4)	543 (17.6)		651 (21.7)	669 (18.0)	
High school	892 (24.5)	733 (23.8)		776 (25.9)	849 (22.8)	
Apprenticeship	1314 (36.1)	1063 (34.5)		1054 (35.2)	1323 (35.6)	
Mandatory	655 (18.0)	742 (24.1)		518 (17.3)	879 (23.6)	
Smoking (%)			0.052			0.271
Never	1453 (39.9)	1279 (41.4)		1207 (40.3)	1525 (40.9)	
Former	1227 (33.7)	956 (31.0)		1003 (33.4)	1180 (31.7)	
Current	958 (26.3)	854 (27.7)		789 (26.3)	1023 (27.4)	
Physically active (%)	1964 (54.0)	1543 (51.4)	0.037	1594 (53.2)	1913 (52.6)	0.628

Results are expressed as average ± standard deviation or as number of participants and (percentage). Between-group comparisons performed using t-test for continuous variables and chi-square for categorical variables.

Supplementary table S2: comparison of the characteristics at baseline (2003-2006)-of participants who completed the first and the second follow-ups, according to gender, CoLaus|PsyCoLaus study, Lausanne.

	5-year			10-year		
	**Women**	**Men**	**P-value**	**Women**	**Men**	**P-value**
Sample size	**1724**	**1914**		**1373**	**1626**	
Age (years)	51.6 ± 10.5	51.6 ± 10.4	0.992	51.0 ± 10.6	50.5 ± 10.1	0.206
Born in Switzerland (%)	1082 (62.8)	1208 (63.1)	0.826	858 (62.5)	1022 (62.9)	0.838
Education (%)			<0.001			<0.001
University	297 (17.2)	480 (25.1)		244 (17.8)	407 (25.0)	
High school	443 (25.7)	449 (23.5)		373 (27.2)	403 (24.8)	
Apprenticeship	612 (35.5)	702 (36.7)		459 (33.4)	595 (36.6)	
Mandatory	372 (21.6)	283 (14.8)		297 (21.6)	221 (13.6)	
Smoking (%)			<0.001			<0.001
Never	802 (46.5)	651 (34.0)		652 (47.5)	555 (34.1)	
Former	479 (27.8)	748 (39.1)		383 (27.9)	620 (38.1)	
Current	443 (25.7)	515 (26.9)		338 (24.6)	451 (27.7)	
Physically active (%)	959 (55.6)	1005 (52.5)	0.060	741 (54.0)	853 (52.5)	0.409
BMI (kg/m^2)	24.8 ± 4.7	26.3 ± 3.6	<0.001	24.9 ± 4.6	26.1 ± 3.6	<0.001
BMI categories (%)			<0.001			<0.001
Underweight	41 (2.4)	11 (0.6)		29 (2.1)	9 (0.6)	
Normal	1005 (58.3)	745 (38.9)		782 (57.0)	664 (40.8)	
Overweight	457 (26.5)	906 (47.3)		390 (28.4)	742 (45.6)	
Obese	221 (12.8)	252 (13.2)		172 (12.5)	211 (13.0)	
Waist (cm)	82.6 ± 12.1	94.7 ± 10.5	<0.001	82.7 ± 11.8	94.2 ± 10.5	<0.001
Abdominal obesity (%)	516 (29.9)	440 (23.0)	<0.001	418 (30.4)	355 (21.8)	<0.001
Vitamin D levels (nmol/L)	47.0 ± 21.5	46.8 ± 22.0	0.817	47.8 ± 21.8	47.5 ± 22.2	0.662
Vitamin D categories (%)			0.383			0.387
Normal	179 (10.4)	217 (11.3)		153 (11.1)	196 (12.1)	
Insufficiency	541 (31.4)	565 (29.5)		437 (31.8)	482 (29.6)	

Deficiency	1004 (58.2)	1132 (59.1)		783 (57)	948 (58.3)	

Results are expressed as average ± standard deviation or as number of participants and (percentage). Between-group comparisons performed using t-test for continuous variables and chi-square for categorical variables.
